# Inherent Dynamics of Head Domain Correlates with ATP-Recognition of P2X4 Receptors: Insights Gained from Molecular Simulations

**DOI:** 10.1371/journal.pone.0097528

**Published:** 2014-05-30

**Authors:** Li-Dong Huang, Ying-Zhe Fan, Yun Tian, Yang Yang, Yan Liu, Jin Wang, Wen-Shan Zhao, Wen-Chao Zhou, Xiao-Yang Cheng, Peng Cao, Xiang-Yang Lu, Ye Yu

**Affiliations:** 1 Department of Pharmacology and Institute of Medical Sciences, Shanghai Jiao Tong University School of Medicine, Shanghai, China; 2 College of Bioscience and Biotechnology, Hunan Agricultural University, Changsha, China; 3 Putuo District Center Hospital, Shanghai University of Chinese Traditional Medicine, Shanghai, China; 4 Jiangsu Province Institute of Traditional Chinese Medicine, Nanjing, Jiangsu, China; Oak Ridge National Laboratory, United States of America

## Abstract

P2X receptors are ATP-gated ion channels involved in many physiological functions, and determination of ATP-recognition (AR) of P2X receptors will promote the development of new therapeutic agents for pain, inflammation, bladder dysfunction and osteoporosis. Recent crystal structures of the zebrafish P2**X**4 (zfP2**X**4) receptor reveal a large ATP-binding pocket (ABP) located at the subunit interface of zfP2**X**4 receptors, which is occupied by a conspicuous cluster of basic residues to recognize triphosphate moiety of ATP. Using the engineered affinity labeling and molecular modeling, at least three sites (S1, S2 and S3) within ABP have been identified that are able to recognize the adenine ring of ATP, implying the existence of at least three distinct AR modes in ABP. The open crystal structure of zfP2**X**4 confirms one of three AR modes (named AR1), in which the adenine ring of ATP is buried into site S1 while the triphosphate moiety interacts with clustered basic residues. Why architecture of ABP favors AR1 not the other two AR modes still remains unexplored. Here, we examine the potential role of inherent dynamics of head domain, a domain involved in ABP formation, in AR determinant of P2**X**4 receptors. *In silico* docking and binding free energy calculation revealed comparable characters of three distinct AR modes. Inherent dynamics of head domain, especially the downward motion favors the preference of ABP for AR1 rather than AR2 and AR3. Along with the downward motion of head domain, the closing movement of loop_139–146_ and loop_169–183,_ and structural rearrangements of K70, K72, R298 and R143 enabled ABP to discriminate AR1 from other AR modes. Our observations suggest the essential role of head domain dynamics in determining AR of P2**X**4 receptors, allowing evaluation of new strategies aimed at developing specific blockers/allosteric modulators by preventing the dynamics of head domain associated with both AR and channel activation of P2**X**4 receptors.

## Introduction

P2X receptors, ion channels activated by extracellular ATP and permeable to Na^+^, K^+^ and Ca^2+^, are involved in a large array of physiological functions[1] including presynaptic modulation[2], [3], [4], synaptic transmission[5], [6], smooth muscle contraction[7], [8], inflammation[9], [10], [11], [12], cancer[13], intestinal motility[14], [15], taste[16], pain[17], [18], [8], [10], and the regulation of immune[19] and cardiovascular[20], [21] responses, and therefore, are potential therapeutic targets of many diseases [22], [23]. Today, seven genes have been identified to encode distinct P2X subunit isoforms, denoted P2X1 to P2X7, with functional P2X receptors formed as homotrimers or heterotrimers of those subunit isoforms [24]. All P2X subunits share a common topology, characterized with a large, glycosylated, disulphide-rich extracellular (EC) loop, two transmembrane (TM) domains and intracellular N- and C- termini[25], [26], [24]. ATP binds to extracellular subunit interfaces and evokes a conformational change of P2X receptor, leading to the opening of non-selective channel pore formed by TM domains [25], [26]. Notably, the seven homomeric P2X receptors differ considerably in their biophysical and pharmacological properties, and the formation of heteromeric P2X receptors further confers the divergence in channel properties [24].

Determination of ATP-recognition (AR) is a prerequisite for better understanding physiological and pharmacological functions of these homo- or hetero-trimeric P2X receptors [24]. The EC loop of P2X receptors has ∼280 amino acids, containing no known consensus sequences for agonist binding identified in other ATP-sensitive protein [26], [24], [27]. Experiments on chimeric receptors have narrowed down the region of the ATP-binding sites from K67 to K313 (rat P2**X**4 receptor numbering) [24]. Using alanine-scanning mutagenesis, further studies identified eight residues having the potential to participate in the formation of the ATP binding site: K67, K69, F185, T186, N293, F294, R295 and K313 (rat P2**X**4 numbering). The positively charged lysines coordinate the binding of the negatively charged triphosphate of ATP, while aromatic phenylalanine residues could coordinate the binding of the ATP adenine ring. The open structure of zebra fish P2**X**4 (zfP2**X**4) receptors partially confirmed this predicted AR mode of P2X receptors [26].

Besides the AR mode described above (we called it AR1), at least two other AR modes may take place when ABP is exposed to a certain concentration of ATP. Jiang *et al.* have located ATP-binding sites in rat P2X2 (rP2X2) receptor using an engineered affinity labeling approach [28]. They reported two previously unidentified residues N140 and L186 (rP2X2 numbering) from two adjacent subunits separated by about 18 Å in the rP2X2 homology model at the resting state, suggesting the existence of at least two distinct AR modes [28]. One shows similar features to AR1, while the other mode exhibits distinct characters. Additionally, numerous *in silico* docking studies supported the presence of a third AR mode [24], [29], in which the adenine ring of ATP is deeply buried into the hydrophobic region at the interface of rigid lower body domains from two subunits. All of these observations imply that ABP recognizes ATP through different modes. However, why only AR1 can trigger P2**X**4 channel activation and how the architecture of ABP favors the occurrence of AR1 rather than other AR modes still remain uncertain. Further investigation on the underlying mechanism of the AR determining will help to understand the ligand recognition of these ATP-gated trimeric ion channels.

Taking advantage of the resolved crystal structures of zfP2**X**4, here we then study the structural determinant of AR of P2**X**4 receptors using a diversity of computational approaches, including *in silico* docking, molecular dynamics (MD) simulations, normal mode analysis (NMA), conformational sampling and binding free energy (*Δ*G_bind_) calculations. Our results reveal a potential role of inherent dynamics of head domain in AR determining of P2**X**4 receptors.

## Results

### Three Distinct ATP-recognition Modes of zfP2X4 Receptor: AR1, 2 and 3

**Figure 1:**
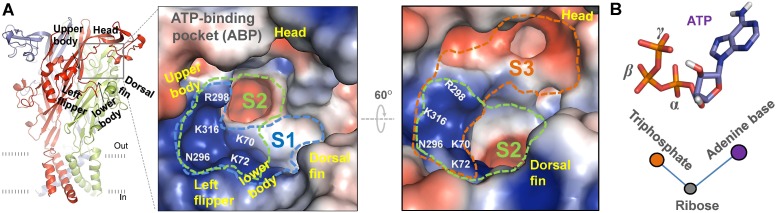
Stereo view of three-dimensional (3-D) structures of zf**P2**X4 and ATP-binding pocket (ABP). (**A**) 3-D architecture of zfP2**X**4 and ATP-binding pocket (ABP). The structure model constructed on the basis of the X-ray crystal closed structure of zfP2**X**4 (PDB entry code: 3H9V) is shown parallel to the membrane layer. Subunits A, B and C are displayed in red, green and blue cartoon, respectively. This figure and following Figures 2–6 are made with PyMol (http://www.pymol.org). 3-D architecture of ABP was highlighted within enlarged square box. S1, 2 and 3 indicate three potential sites that are able to contact with adenine ring of ATP. (**B**) The bent conformation of ATP.

**Figure 2:**
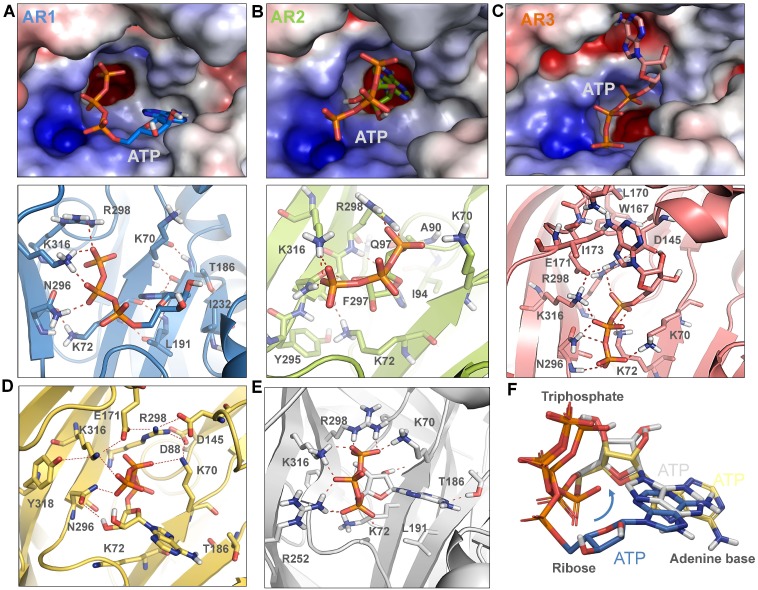
Three distinct potential ATP-recognition modes of zfP2X4 receptors. (**A–C**) Structure and key residues (displayed in sticks for emphasis) of three distinct potential ATP-recognition (AR) modes of zfP2**X**4 receptors: AR1 (**A**), 2(**B**) and 3(**C**). ATP was docked into the potential binding sites, S1, 2 and 3, of zfP2**X**4 using docking program Glide [34]. (**D**) AR1 mode obtained by docking ATP into previously sampled/averaged zfP2**X**4 structure by 2-ns MD simulations. (**E**) AR1 mode obtained by induced fit docking strategy. (**F**) Superposition of ATP poses of AR1 modes obtained by three different strategies, including rigid body docking ATP into static closed structure of zfP2**X**4 (blue) and previously sampled/averaged closed structure of zfP2**X**4 (yellow) by 2-ns MD simulations, and induced fit docking (grey).

The closed structure of zfP2**X**4 receptor reveals a large ATP-binding pocket (ABP) at the interfaces of two subunits (Fig. 1A). ABP is formed by the head and upper body domains of one subunit, and the lower body and dorsal fin domains of another subunit (Fig. 1A). It is widely accepted that the conspicuous cluster of basic and polar residues, R298, K316, K70, K72 and N296, constitute a pocket specific for recognizing the triphosphate moiety of ATP [26]. However, as revealed by closed structure of zfP2**X**4, there are at least three sites in ABP, S1, S2 and S3, can dock the adenine ring of ATP (Fig. 1A). We established those three recognition modes using *in silico* docking (Fig. 2), AR1, 2 and 3, to show initial interactions between ATP and those three sites. In our modes, AR1 had similar features with the open crystal structure of zfP2**X**4 [26], in which the adenine ring made contacts with site S1 (consisting of L191, K70, K72, I232 and T186) (Fig. 2A). For AR2, the adenine ring of ATP is deeply buried into the interface of rigid lower body domains and contacts with I94, F297, Q97 and Y295 (Fig. 2B), a recognition mode proposed by numerous computational studies [29], [24]. AR3 showed similar features revealed by covalent binding of NCS-ATP to engineered cysteine residues in the putative ATP-binding sites of rP2X2 [28], where the adenine ring of ATP contacts with site S3 (W167, I173, L170, D145 and E171) (Fig. 2C).

We further characterized these three distinct AR modes through additional computational approaches. It has been reported that ATP may bend into ‘U’ or ‘V’-shaped structure (Fig. 1B) with the *β*- and *γ*-phosphates folded towards the adenine ring, as observed in ATP-bound opening zfP2**X**4 [26] and class II aminoacyl transfer RNA synthetases[30]. Our *in silico* docking studies showed that the bound ATP could adopt bent conformations when adenine ring of ATP made contacts with any one of those three sites despite the differences in the bending angle (Fig. 2A–C), suggesting that sites S1, 2 and 3, together with the charged region, are capable to fit bended ATP. Additionally, as revealed by combining the docking and *Δ*G_bind_ calculations (Table 1), AR2 induced a little more free energy release than AR1 did (*Δ*G_bind_ = −33.6±4.1, −43.2±3.9 and −28.8±4.4 kcal/mol for AR1, AR2 and AR3, respectively), indicating higher ATP-affinities of AR2 than AR1, at least in molecular simulations. Thus, the *Δ*G_bind_ analysis does not support the dominant presence of AR1. Additionally, ATP was also docked into the closed structure of zfP2**X**4 that has been previously sampled and averaged on short timescale (2-ns) MD simulations (Fig. 2D, F), and an induced fit docking strategy was also performed on this closed structure (Fig. 2E, F). *Δ*G_bind_ calculations indicated that those two docking strategies still did not support the dominant presence of AR1 (Table 1), implying that more dynamic features in ABP are responsible for the dominance of AR1 over AR2 and 3.

**Table 1:** Summary of the MM-GBSA calculations of binding free energy of ATP (*Δ*G_bind_) based on the proposed ATP-binding modes.

Protein structures#	Docking	ATP-binding(kcal/mol)	MM/GBSAΔG bind&	ΔG bind Coulomb	ΔG bind vdW	ΔG bind Solv	Ligand strain energy	Receptor strain energy
Closed structure	Rigid body docking^a^	AR1	−33.6±4.1	−117.1±8.3	−42.6±1.1	113.3±8.9	3.09±0.41	9.91±2.50
		AR2	−43.2±3.9	−115.6±1.3	−48.0±2.6	118.6±6.5	10.1±0.41	−8.41±2.02
		AR3	−28.8±4.4	−140.4±26.3	−48.6±2.5	144.6±8.6	7.37±2.48	7.82±2.13
	Rigid body docking^b^	AR1	−4.13±1.41	−83.0±7.1	−50.5±1.0	115.7±9.3	10.3±0.22	3.40±2.77
		AR2	−50.8±10.0	−66.2±8.2	−51.9±0.6	61.15±1.9	5.20±0.06	1.07±4.71
		AR3	−6.45±1.92	−122.8±6.3	−38.9±0.1	142.3±8.6	8.60±0.09	4.36±0.21
	Induced fit docking	AR1	−20.85±1.30	−141.7±7.1	−45.2±1.0	141.5±1.2	5.98±0.10	18.58±1.31
		AR2	−21.13±4.92	−128.1±1.2	−41.3±3.4	128.2±1.0	10.6±0.81	9.47±2.10
		AR3	−19.25±7.20	−115.6±0.8	−50.5±0.5	126.4±1.4	8.98±0.14	11.52±7.18
Open structure	Rigid body docking^a^	AR1	−46.79±7.7	−263.6±15.6	−42.8±0.5	240.2±5.5	5.82±0.78	13.58±2.0
		AR2	n.d.	n.d.	n.d.	n.d.	n.d.	n.d.
		AR3	−27.52±5.04	−224.6±16.3	−44.6±1.5	219.2±10.7	8.94±0.24	12.76±3.8

# the PDB entry codes for closed and open structures used in *in silico* docking are 3H9V and 4WD1, respectively;

a Docking ATP into closed or open structures of zfP2**X**4.1 with proper preparation and minimization beforehand through a rigid body docking strategy;

b Docking ATP into closed structure of zfP2**X**4.1 that has been previously sampled and averaged using 2-ns MD simulations through a rigid body docking strategy;

& *Δ*G_bind_-Coulomb, *Δ*G_bind_-vdW, *Δ*G_bind_-Solv represents contribution of Coulomb energy, van der Waals energy and Generalized Born electrostatic solvation energy in *Δ*G_bind_ calculation, respectively. An estimate of strain energies for the ligand and the receptor were also shown in summary.

*n.d.*, the *Δ*G_bind_ value of AR2 was not measured for there is no AR2 pose observed in *in silico* docking.

### Inherent Dynamics of Head Domain is Able to Change the Preference of ABP for AR1, 2 and 3

**Figure 3:**
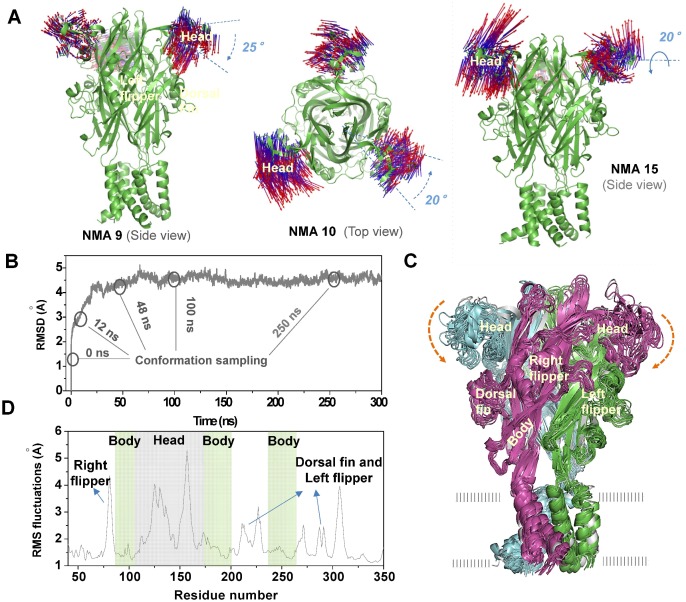
General motions of head domain detected by NMA and MD simulations. (**A**) General motions of the head domain in NMA modes 1 (left), 2 (middle) and 3 (right). The vectors representing both the amplitudes and directions of the displacements experienced by residues during the conformation changes are mapped onto the head domain. These three modes were derived from low-frequency modes NMA 9, 10 and 15 during NMA analysis (http://lorentz.immstr.pasteur.fr/nomad-ref.php), respectively. As indicated by the vectors, the motions can be described approximately as a downward motion (mode 1), a rotation around the central axis of protein (mode 2), and a rotation around the central axis of itself (mode 3). Blue arrows show the directions of motion of head domain. (**B**) Time-dependence of the root mean square deviation (RMSD) of the C_α_ atoms from the initial zfP2**X**4 structure in MD simulations. The average conformations around initial (0 ns), 12, 24, 48, 100 and 250 ns were sampled to performed further *in silico* docking. (**C**) Spontaneous downward motion of head domain during MD simulations. 13 snapshots were superimposed to the initial structure (grey) by fitting all the C_α_ atoms of the zfP2**X**4. The arrows show the direction of motions. (**D**) r.m.s. fluctuations of various domains during MD simulations.

As revealed by crystal structures of zfP2**X**4, both closed and open structures, head domain exhibits higher B-factor values than other domains involved in forming ABP[26], such as the lower body domain and dorsal fin domain (Fig. 1A). Previous studies have demonstrated the critical role of head dynamics in channel activations [31], [32]. Here, taking account of its role in ABP formation, we proposed that the dynamics of head domain would affect the recognition of ATP. To test this hypothesis, we extensively investigated the relationship between the inherent dynamics of head domain and AR of P2X receptors using MD, NMA and *in silico* docking. As revealed by low frequency modes of NMA, head domain displayed three possible dynamic modes (Fig. 3A): a downward motion, a rotation around the central axis of itself, and a rotation around the central axis of protein. MD simulations confirmed a more notable conformational fluctuation of head domain than that of body, left flipper, right flipper and dorsal fin domains in the absence of ATP (Fig3. B–D). Moreover, a spontaneous downward motion of head domain was observed in MD simulations (Fig. 3C), indicating the preference of zfP2**X**4 to the downward motion of head domain even without ATP binding. In open structure of zfP2**X**4, ATP binding induces slightly downward motions of the head domain and subsequently closing of ATP-binding jaw [26], confirming the idea that the downward motion may be the inherent dynamics of head domains.

**Figure 4:**
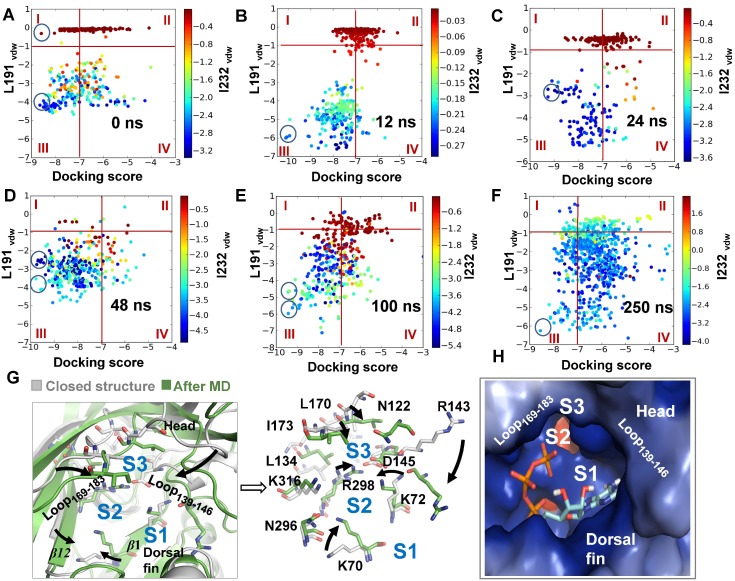
Molecular docking of ATP into various sampled conformations of zf**P2**X4. (**A–F**) Summary of poses in various sampled conformations of zfP2**X**4. These conformations were sampled from snapshots of MD simulations, during which downward motion of head domain occurred. In this panel and Figure 6B, the poses distributed into region I and III represent rational recognition modes of ATP, AR2/3 and AR1, respectively. The docking scores and *vdW* scores of L191 and I232 of per poses were calculated by Glide [34]. Each dot represents a pose of ATP. The colors of dot indicate the *vdW* scores of I232. Blue dot means strong *vdW* contacts between ATP and I232. The top poses of each sampled conformation of receptors were highlighted within black cycles. (**G**) Superposition of the initial structure (grey) and equilibrated representative structure (green) after 300-ns MD simulations. Black arrows denote the movements of key domains/regions and residues (displayed in sticks for emphasis) from the initial structure to equilibrated structure. (**H**) 3-D architectures of ABP with docked-ATP in the equilibrated representative structure of zfP2**X**4 after MD simulations. S1, S2 and S3 indicate three potential sites interacting with the adenine ring of ATP.

To further test the contribution of head domain dynamics to determination of AR in P2**X**4, six representative conformations were sampled by getting the ‘average’ conformations around 0, 12, 24, 48, 100, and 250 ns upon the initiation of spontaneous downward motion of head domain during MD simulations (Fig. 3B, D). ATP was docked into the sampled conformations of zfP2**X**4, and various ATP-poses were then characterized and plotted according to their total docking scores and *vdW* scores of ATP contacting with L191 (L191_vdW_) and I232 (I232_vdW_) (see Fig. 4A–F, each colorful dot represents an ATP pose). Poses with lower values of docking score (<−7), and lower values of L191_vdW_ and I232_vdW_ scores (<−1) (region III, Fig. 4A–F) were regarded as rational AR1 mode in which the adenine ring of ATP makes a contact with L191, I232 and nearby hydrophobic residues while the triphosphate moiety contacts with clustered basic residues. On the other hand, lower values of docking score (<−7), and higher values of L191_vdW_ and I232_vdW_ scores (>−1) (region I in Fig. 4A–F) indicated that the adenine ring of ATP makes no contacts with these two amino acid, reflecting rational AR2 or 3 modes. Along with the motion of head domain, the top ATP poses with the lowest docking score within region I (AR2 and 3) and III (AR1) were obviously altered (Fig. 4A–F, within the highlighted circles). For initial conformation of zfP2**X**4 (0 ns), the top ATP poses distributed both in region I and III showed comparable lowest docking scores (Fig. 4A). However, the top ATP poses only occurred within region III instead of region I (Fig. 4B–F) along with the downward motion of head domain, indicating that this motion favors the AR1’s occurrence rather than AR2 and 3. These data suggested that inherent conformational fluctuations of head domain, especially the downward motion, can alter the preference of ABP for AR1, 2 and 3.

A superposition of closed structure and equilibrated representative structure after 300-ns MD simulations showed that the downward motion of head domain was followed by a marked change in the orientation of R143 side chain, a leftward movement of loop_139–146_ (a region within the head domains) and a rightward movement of loop_169–183_ (a region covalently linking with the head domains). The closing movement between these two loop regions dramatically changed the shape of site S3 (Fig. 4G, H), which prevented ATP from contacting with site S3. It also brought the basic residues K70, K72 and R298 as well as *β*1 and *β*12 close to each other (Fig. 4G), which significantly narrowed entrance of ATP into site S2 (Fig. 4G, H) and prevented contacts between the adenine ring of ATP and site S2. On the contrary, the downward motion of R143 and rightward movement of loop_139–146_ facilitated the contacts between ATP and site S1 via a mechanism of partially trapping adenine ring of ATP into the narrowed cleft formed by loop_139–146_ and dorsal fin domain (Fig. 4G, H). Meanwhile, the altered orientations of K70, K72 and R298 would favored coulombic interactions between triphosphate moiety of ATP and basic groups of those three residues. These allosteric changes resulted in an increased preference of ABP for AR1 and prevent ATP from interacting with sites S2 and S3.

### AR1 Instead of AR2 and 3 Favors the Downward Motion of Head Domain and Downstream Allosteric Changes Associated with the Channel Activation of P2X4 Receptors

**Figure 5:**
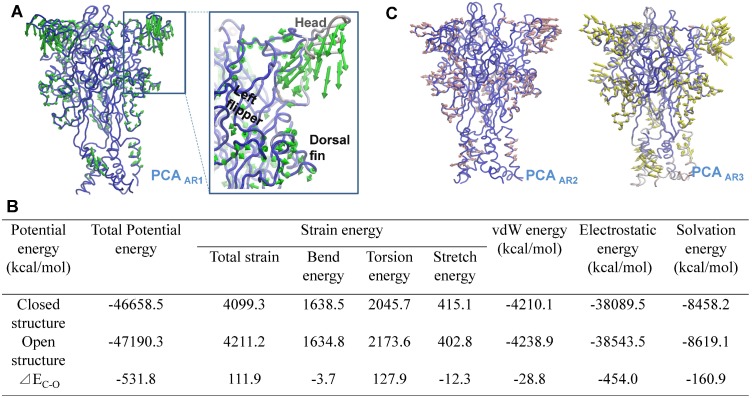
AR1’s occurrence-mediated ‘induced-fit’ allosteric changes in ABP and whole zf**P2**X4 receptor (**A**) Vector representation of the motions of zfP2**X**4 (PDB entry code: 3H9V) in principal component analysis (PCA) based on the snapshots from 10-ns MD simulation of zfP2**X**4-ATP complex in AR1 mode. The vector arrows represent both the amplitudes and directions of the displacements experienced by residues during conformational changes. (**B**) Summary of the potential energy of receptors at the resting and open states. (**C**) Vector representation of the motions of zfP2**X**4 in PCA analysis based on the snapshots from 10-ns MD simulation of zfP2**X**4 complexes with pre-docked ATP based on AR2 and AR3 modes.

We also found that AR1 rather than AR2 and 3 can promote the inherent downward motion of head domain (Fig. 5A). A combination of 10-ns MD simulations of and PCA analysis of snapshots from simulations revealed that AR1-induced a downward movement of the head domain, upward motion of dorsal fin domain and a subsequent closing of ATP-binding site jaw (Fig. 5A), consistent with previous observations[31], [26]. Meanwhile, it has been well established that those allosteric changes are associated with downstream opening-related allosteric changes, such as the radial expansions of extracellular vestibule and the final iris-like channel opening [26], [33]. The potential energy calculations on resting and open states of zfP2**X**4 receptors revealed that AR1-induced allosteric changes led to energy releases during conformational transition between the resting and open states (Fig. 5B). In contrast, MD simulations together with PCA analysis showed that AR2 and 3 can only induce outward and upward movements of head domain, respectively (Fig. 5C). Therefore, AR2 and AR3 would not trigger downstream allosteric changes. These observations provided a conceivable explanation for why only AR1 rather than AR2 and 3 is able to trigger channel activation of P2**X**4 receptors. The *Δ*G_bind_ together with the downstream allosteric changes-induced energy releases may enable AR1 to efficiently overcome the energy barrier for the channel gating.

**Figure 6:**
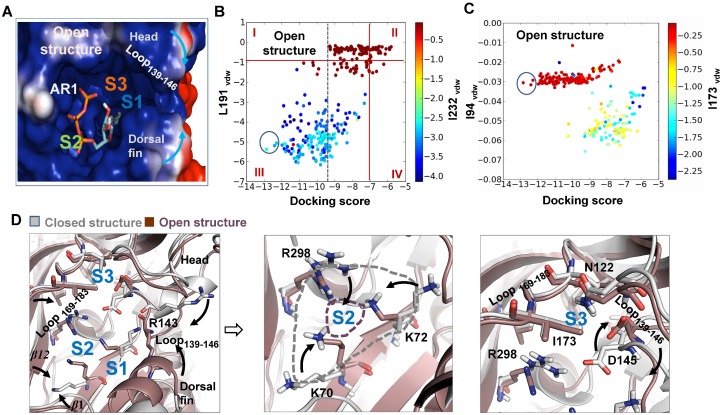
AR1’s occurrence gives rise to a decreased preference of ABP for AR2 and 3. (**A**) AR1 mode in the open structure (PDB entry code: 4DW1). S1, S2 and S3 indicate three potential sites contacting with the adenine ring of ATP. (**B–C**) Summary of poses in opening structure of zfP2**X**4. The docking scores and *vdW* scores of L191, I232, I94 and I232 of per poses were calculated by Glide [34]. Each dot represents a pose of ATP. The colors of dot indicate *vdW* scores of I232 (**B**) or I173 (**C**). The blue dot means strong *vdW* contacts between ATP and L191 (**B**) or I173 (**C**). The top poses of each sampled conformation of receptors were highlighted within cycles. (**D**) Superposition of closed (grey, PDB entry code: 3H9V) and open structures (light brown, PDB entry code: 4DW1). Black arrows denote the movements of key domains/regions and residues (displayed in sticks for emphasis) from the resting to open structures. The changed sizes of site S2 in closed (grey) and open (brown) structures are highlighted with dotted lines.

Meanwhile, based on the observation that conformational fluctuations of head domain, especially the downward motion, greatly enhance the preference of ABP for AR1 (Fig. 4A–F), we proposed that the downward motion of head domain facilitated by AR1 may further increase the preference of ABP for AR1. It looks like an induced-fit/positive feedback mechanism when AR1 occurs. Indeed, when we compare conformations of resting sate and the open state as well as their corresponding *Δ*G_bind_ values of ATP, AR1-induced downward movement of the head domain, upward motion of dorsal fin domain and the closing of ATP-binding site jaw in open state (Fig. 6A) led to significant increase in free energy release than that in resting state (*Δ*G_bind_ of ATP = −33.6±4.1 and −46.79±7.7 kcal/mol, for resting and open states, respectively, Table 1). This increased free energy release upon ATP-binding may attribute to the dynamics of head domain together with dorsal fin upward movement-induced partial trapping of the adenine ring of ATP into site S1 of ABP (Fig. 6A).

Taken together, the contact between ATP and site S1 (AR1) promoted downward motion of head domain correlated well with conformational changes of ABP and subsequently increased preference of ABP for AR1. Once ATP comes in contact with site S1, it will initiate an ‘induced-fit’/positive-feedback mechanism, with the increased free energy release and the downstream allosteric changes-induced energy releases acting as the driving force. In contrast, AR2 and 3 are not capable of facilitating the downward motion of head domain and therefore, would not evoke such positive feedback.

### The Allosteric Changes of Head Domain Induced by AR1 Attenuate the Preference of ABP for AR2 and 3

**Figure 7:**
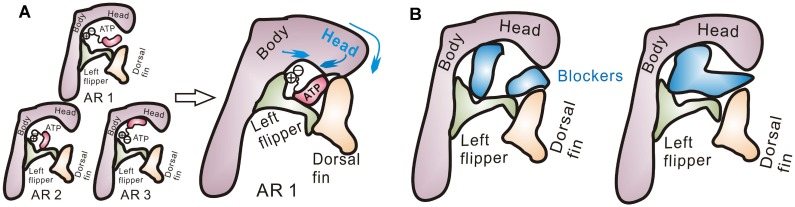
Illustrations of allosteric changes associated with AR determining and strategies to *de novo* design new blockers of P2X4 receptors. (**A**) Allosteric changes associated with zfP2**X**4 discriminating different AR modes. Light blue arrows denote the movements of key domains/regions crucial for AR determinants. (**B**) Illustrations of strategies to *de novo* design new blockers of P2**X**4 receptors.

Beside the positive-feedback mechanism of AR1 on itself, the possibility that AR1 exerted a negative effect of AR1 on the occurrence of AR2 and 3 is also examined here. Taken into consideration of the involvement of head domain in forming S2 and S3, AR1-induced dynamics of head domain may also change the preference of ABP for AR2 and 3 accompanying the AR1’s occurrence. To test this idea, ATP was further docked into ABP of open crystal structure of zfP2**X**4 (Fig. 6A, B), a state representing the stable conformation of AR1-induced alterations both in head domain and ABP. Interestingly, the top ATP poses with very low values of docking scores (<−12) and low values of L191_vdW_ and I232_vdW_ scores can be easily observed in region III (Fig. 6B), confirming the ‘induced-fit’ mechanism of AR1 on itself. In contrast, as revealed by the approaching zero scores of I94_vdW_ in all the ATP poses (Fig. 6C), no poses possessing features of AR2 occurred during *in silico* docking. This result indicated that AR1-induced conformation changes of ABP preclude the contact between ATP and site S2. The I173_vdW_ scores revealed that a few poses possessing features of AR3 can be also observed in molecular docking (Fig. 6C, blue dots). However, AR3-induced free energy release was much less than that of AR1 (*Δ*G_bind_ = −46.79±7.7 and −27.52±5.04 kcal/mol, for AR1 and 3 in open state, respectively, Table 1). These results suggested that AR1-induced allosteric change of head domain would decrease the preference of ABP for AR2 and 3. The positive feedback of AR1 on itself together with the negative effect it exerted on AR2 and 3 will dramatically improve the ability of ABP for AR1 recognition. Notably, along with the downward motion of head domain, the closing movement of loop_139–146_ and loop_169–183_ and structural rearrangements of K70, K72, R298, R143 and D145 enable ABP in the open structure to discriminate AR1 from other AR modes. Consistent with the MD data (Fig. 4G), our results support the pivotal role of the conformational changes of head domain in AR determinants (Fig. 7A).

### Discussion and Conclusions

Agonist-recognition is a fundamental question for ligand-gated ion channels. Although the newly resolved crystal structures of zfP2**X**4 uncover exact AR mode (AR1) of these trimeric ion channels [26], it remains unclear why ABP of P2X receptor favors AR1 while other AR modes proposed by both experimental and computational approaches cannot trigger the activation of those receptors. Understanding the underlying mechanism of AR will greatly contribute to the development of novel antagonists/allosteric modulators of P2X receptors [24]. Using a diversity of computational approaches, we suggested that the following factors may contribute to the preference of ABP for AR1 and abilities of AR1 rather AR2 and 3 in P2**X**4 receptors gating: *First,* site S1 is located on the surface of P2X receptor while S2 and 3 are partially buried by head domain and body domain (Fig. 1A), thus, the probability of ATP contacting with site S1 is a little higher than that of S2 and S3. *Secondly,* although the binding free energy releases of three distinct AR modes at the very beginning of ATP contacting with resting P2**X**4 receptors were comparable, the increased free energy release, springing from AR1-mediated promoted downward motion head domain, together with the downstream allosteric changes-induced significantly increased energy releases, which would enable AR1 to overcome the energy barrier required for channel gating. In contrast, AR2 and 3 would preclude the downward motion of head domain and the downstream allosteric changes correlated with channel gating (Fig. 5C). *Third,* beside the positive feedback of AR1 on itself, AR1’s occurrences-induced allosteric changes of ABP is able to preclude the occurrence of AR2 and 3(Figs. 4G, H and 6D). This idea is supported by the observation that no AR2 poses was observed during docking of ATP into open conformation of zfP2**X**4 (Fig. 6C). All of these contribute to the preference of ABP for AR1 over AR2 and 3 during channel gating.

The comparisons between closed and open structures, and the equilibrated averaged structure after MD simulations would not only facilitate studying ligand-recognition of P2**X**4 receptors (Fig. 7A), but also will provide some clues to understanding of channel gating mechanism. The movements observed in MD simulations exhibited high a similarity to bound-ATP induced conformational changes of ABP in open crystal structure. For instance, the structural rearrangements of K70, K68, R289 and R143, and closing movements of loop_139–146_ and loop_169–183_ (Figs. 4G, H and 6D). However, the rightward movement of loop_169–183_ and the down movement of head domain during MD simulations were more evident than those of ATP-bound open structure (Figs. 4G, H and 6D). On the contrary, the upward movement of head domain in MD data was not as obvious as that of open structure (Figs. 4G and 6D). These observations confirmed the crucial role of inherent dynamics of head domain in both AR and channel gating of P2**X**4 receptors. However, the complex dynamics in the process of channel gating well beyond the inherent dynamics of head domain, during which bound-ATP evoked coordinated movements of multiple domains, especially for the head and dorsal fin domains, are crucial for this process.

Our findings also provide new strategies for developing specific blockers/allosteric modulators by preventing the dynamics of head domain associated both with AR and channels activation of P2**X**4 receptors (Fig. 7B). Three types of new molecules could interrupt P2**X**4 activation based on our findings: *First,* we can *de novo* design high affinity small molecules containing both the acidic group to interact with K70, 72, K316, K193, R298 and the heterocyclic group to finely match the shape of site 2 or 3 (Fig. 7B). These small molecules can compete with ATP and meanwhile act as allosteric modulator to prevent the dynamics of head domain. The second type of molecules are designed to fill up the cleft between the head domain and the dorsal fin domain. This strategy has also been proposed by Jiang *et al.* as inhibitor binds to this position can prevent the closing of ATP-binding site jaw [33], an allosteric change essential for channel activation. Here we predict that small molecules at this position would block the inherent dynamics of head domain. As a result, more ATP would contact with sites S2 and S3. Additionally, ATP contacting with site S1 induced positive-feedback can also be inhibited by these small molecules. Therefore, molecules that filling up the cleft between the head domain and the dorsal fin domain would impede the probability of AR1’ occurrence at the very beginning of ATP contacting with P2**X**4. *Third*, we can design *de novo* small molecules with a bulky size to occupy both the whole ABP and the cleft between head and dorsal fin, which can both compete with ATP and prevent the dynamics of head domain as well as the closing of ATP-binding site jaw.

In summary, our study brings new insights into the mechanism of ATP recognition. The crucial role of head domain dynamics in channel activation has been well established, here we discuss its potential role in determining AR of P2**X**4 receptors. Our findings provide strategies in designing new blockers that prevent the dynamics of head domain associated with both AR and channel activation of P2**X**4 receptors.

## Methods

### *In silico* Docking

The docking program Glide [34] was applied to dock ATP into the potential binding site of closed (PDB entry code: 3H9V) and open (PDB entry code: 4DW1) zfP2**X**4 as our previous description [35]. Briefly, for Glide docking, the zfP2**X**4 structure was preprocessed using the protein preparation and refinement components. Then the grid for the protein was defined as an enclosing cubic box within 30 Å of centroid of selected reside (K70) in ABP of zfP2**X**4, where sites S1, S2 and S3 were all included. Conformations of ATP were generated by LigPrep [36]. For docking runs, the extra precision (XP) docking mode was selected. All of the procedures including protein preparation, refinement, grid generation, and docking were performed using the default parameters except for the parameters for ATP poses output. During *in silico* docking, at most 100000 poses per docking run were selected, among of which top 300 poses per conformation of ATP were performed post-docking minimization. The threshold for rejecting minimized pose is 0.5 kcal/mol. At most 100 poses per conformation of ATP will be finally wrote out. ATP-per residue interaction scores were also measured during *in silico* docking. The docking scores and ATP-residue interaction scores were summarized, sorted and then plotted by Maestro (https://www.schrodinger.com/productpage/14/12/37/). Induced fit docking was performed by Glide^38^. The residues within 15 Å of ligand pose were refined.

### MM-GBSA Binding Free Energy Calculation

Binding free energy (*Δ*G_bind_) calculations were performed by Prime MM-GBSA[37]. The pose files obtained from Glide were the source of structures, where ligands and receptors were properly prepared beforehand. Residues that have any atoms within the 15 Å of the ATP processed are included in the flexible region. The movement of the flexible residues were not constrained with a harmonic potential. The binding energy is calculated according to the equation: *ΔG*_bind_ *= G*_complex_ *−*(*G*_ligand_ *+G*_receptor_).

### Molecular Dynamics Simulations

According to our previous description [38], MD simulations were performed by using the program Desmond 3.0[39] with constant number of particles, constant pressure and temperature, and periodic boundary conditions, which uses a particular “neutral territory” method called the midpoint method to efficiently exploit a high degree of computational parallelism. Briefly, the closed structure of zfP2**X**4 (PDB entry code: 3H9V) or closed structure of zfP2**X**4 (PDB entry code: 3H9V) complexes with pre-docked ATP were used as the starting structures for MD simulations. A large dimyristoylphosphatidylcholine bilayer was constructed to generate a suitable membrane system where the TM domain of the zfP2**X**4 could be embedded. The protein/dimyristoylphosphatidylcholine system was then solvated in a bath of simple point charge water molecules. Counter ions Na^+^ were subsequently added to compensate for the net negative charge of the system. A default OPLS_2005 force field was employed for the protein or protein-ligand complex. To maintain the system at a constant temperature of 300 K, the Berendsen algorithm was applied to couple protein and other molecules separately with a coupling time of 0.1 ps. All of the bond lengths including hydrogen atoms were constrained by the Linear Constraint Solver algorithm. Electrostatic interactions between charged groups at a distance less than 9 Å were calculated explicitly. Long range electrostatic interactions were calculated using the smoothed particle mesh Ewald method. All of the MD simulations were run on the DAWNING TC2600, with 200 AMD Opteron™ 8374HE CPUs). All MD simulations were repeated in at least two independent runs.

### Normal Mode Analysis and PCA Analysis

According to our previous description [40], the atomic coordinates for the crystal structure of zfP2**X**4.1 (PDB entry code: 3H9V) with proper preparation and minimization beforehand was used as the starting structure in a series of computational simulations and calculations. NMA was conducted using the web server developed by Delarue et al. (http://lorentz.immstr.pasteur.fr/nomad-ref.php) [41]. During the NMA simulations, the single-parameter Hookean potential, a simplified all-atom potential [41], was used:
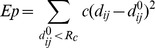
where *d_ij_* is the distance between two atoms *i* and *j*, *d* ^0^ *_ij_* is the distance between the atoms in the three-dimensional structure, *c* is the spring constant of the Hookean potential (assumed to be the same for all interacting pairs) and *R_c_* is an arbitrary cut-off. In this study, *R_c_* was set to be 10 Å. PCA analysis were applied by using the program ProDy 1.1 [42]. PCA modes of zfP2**X**4 dynamics were obtained by essential dynamics analysis (EDA) of snapshots of MD simulations of zfP2**X**4 receptors that ATP has been pre-docked into sites S1, S2 or S3. This measurement was performed using the default Prody parameters. 5 modes were generated in PCA analysis by Prody [42].
